# Characteristics and risk factors for SARS-CoV-2 in children tested in the early phase of the pandemic: a cross-sectional study, Italy, 23 February to 24 May 2020

**DOI:** 10.2807/1560-7917.ES.2021.26.14.2001248

**Published:** 2021-04-08

**Authors:** Marzia Lazzerini, Idanna Sforzi, Sandra Trapani, Paolo Biban, Davide Silvagni, Giovanna Villa, Jessica Tibaldi, Luca Bertacca, Enrico Felici, Giuseppina Perricone, Roberta Parrino, Claudia Gioè, Sara Lega, Mariasole Conte, Federico Marchetti, Annamaria Magista, Paola Berlese, Stefano Martelossi, Francesca Vaienti, Enrico Valletta, Margherita Mauro, Roberto Dall’Amico, Silvia Fasoli, Antonio Gatto, Antonio Chiaretti, Danica Dragovic, Paola Pascolo, Chiara Pilotto, Ilaria Liguoro, Elisabetta Miorin, Francesca Saretta, Gian Luca Trobia, Antonella Di Stefano, Azzurra Orlandi, Fabio Cardinale, Riccardo Lubrano, Alessia Testa, Marco Binotti, Valentina Moressa, Egidio Barbi, Benedetta Armocida, Ilaria Mariani, on behalf of COVID-19 Italian Pediatric Study Network

**Affiliations:** 1 Institute for Maternal and Child Health - IRCCS “Burlo Garofolo”, Trieste, Italy; 2 Department of Pediatric Emergency Medicine and Trauma Center, Meyer Children's University Hospital, Florence, Italy; 3 Department of Health Sciences and Meyer Children's University Hospital, Florence, Italy; 4 Department of Neonatal and Paediatric Critical Care, Verona University Hospital, Verona Italy; 5 Pediatric Emergency Unit, IRCCS Gaslini Children's Hospital, Genoa, Italy; 6 Pediatric Emergency Unit and Department of Pediatric and Neonatology, Misericordia Hospital, Grosseto, Italy; 7 Pediatric and Pediatric Emergency Unit, The Children Hospital, AO SS Antonio e Biagio e Cesare Arrigo, Alessandria, Italy; 8 Pediatria d'Urgenza e Pronto Soccorso P.O.G. Di Cristina, Palermo, Italy; 9 Pediatric Infectious diseases, P.O.G. Di Cristina, Palermo, Italy; 10 Department of Pediatrics, Ravenna Hospital, Ravenna, Italy; 11 Department of Pediatrics, Community Pediatrics, Ravenna, Italy; 12 Department of Pediatrics, Treviso Hospital, Treviso, Italy; 13 Department of Pediatrics, G.B. Morgagni-L. Pierantoni Hospital, Forlì, Italy; 14 Department of Pediatrics and Neonatology, Santa Maria degli Angeli Hospital, Pordenone, Italy; 15 Paediatric Unit, Carlo Poma Hospital, Mantua, Italy; 16 Department of Woman and Child Health and Public Health, Fondazione Policlinico Universitario A. Gemelli IRCCS, Rome, Italy; 17 Department of Pediatrics, San Polo Hospital, ASUGI, Monfalcone (GO), Italy; 18 Division of Paediatrics, Department of Medicine DAME, Academic Hospital Santa Maria della Misericordia, University of Udine, Udine, Italy; 19 Department of Pediatrics, Latisana-Palmanova, ASUFC, Udine, Italy; 20 Pediatric and Pediatric Emergency Room Unit Cannizzaro Emergency Hospital, Catania, Italy; 21 Giovanni XXIII Pediatric Hospital, Department of Pediatrics, University of Bari, Bari, Italy; 22 Department of Pediatrics Sapienza University of Rome, Santa Maria Goretti Hospital, Latina, Italy; 23 Neonatal and Pediatric Intensive Care Unit, Maggiore della Carità University Hospital, Novara, Italy; 24 University of Trieste, Trieste, Italy; 25 Members of the COVID-19 Italian Pediatric Study Network are acknowledged at the end of the article

**Keywords:** COVID-19, children, Italy, risk factors

## Abstract

**Background:**

Very few studies describe factors associated with COVID-19 diagnosis in children.

**Aim:**

We here describe characteristics and risk factors for COVID-19 diagnosis in children tested in 20 paediatric centres across Italy.

**Methods:**

We included cases aged 0–18 years tested between 23 February and 24 May 2020. Our primary analysis focused on children tested because of symptoms/signs suggestive of COVID-19.

**Results:**

Among 2,494 children tested, 2,148 (86.1%) had symptoms suggestive of COVID-19. Clinical presentation of confirmed COVID-19 cases included besides fever (82.4%) and respiratory signs or symptoms (60.4%) also gastrointestinal (18.2%), neurological (18.9%), cutaneous (3.8%) and other unspecific influenza-like presentations (17.8%). In multivariate analysis, factors significantly associated with SARS-CoV-2 positivity were: exposure history (adjusted odds ratio (AOR): 39.83; 95% confidence interval (CI): 17.52–90.55; p < 0.0001), cardiac disease (AOR: 3.10; 95% CI: 1.19–5.02; p < 0.0001), fever (AOR: 3.05%; 95% CI: 1.67–5.58; p = 0.0003) and anosmia/ageusia (AOR: 4.08; 95% CI: 1.69–9.84; p = 0.002). Among 190 (7.6%) children positive for SARS-CoV-2, only four (2.1%) required respiratory support and two (1.1%) were admitted to intensive care; all recovered.

**Conclusion:**

Recommendations for SARS-CoV-2 testing in children should consider the evidence of broader clinical features. Exposure history, fever and anosmia/ageusia are strong risk factors in children for positive SARS-CoV-2 testing, while other symptoms did not help discriminate positive from negative individuals. This study confirms that COVID-19 was a mild disease in the general paediatric population in Italy. Further studies are needed to understand risk, clinical spectrum and outcomes of COVID-19 in children with pre-existing conditions.

## Introduction

The pandemic of severe acute respiratory syndrome coronavirus 2 (SARS-CoV-2) affected Italy as first country in Europe [1]. The Italian government declared a state of emergency on 31 January 2020 [2] and by 24 May 2020, a total of 229,858 cases of COVID-19 had been diagnosed across the country [3].

From the very beginning of the pandemic, data suggested that children are less affected than adults by COVID-19 [4-10]. However, timely diagnosis of SARS-CoV-2 infection is not only important for the single individual, it is crucial to prevent the spread of the pandemic. A better understanding of the predictors of a positive SARS-CoV-2 test results may facilitate timely case finding and contact tracing and thus contribute to control the pandemic. It may also improve organisation of care in settings where diagnostic facilities are available but still require a considerable processing time, where diagnostic facilities are lacking and where diagnosis, in the absence of other tools, may need to be based on clinical characteristics alone.

Several systematic reviews have synthetised the clinical features and outcomes of paediatric cases with a confirmed SARS-CoV-2 infection [11-17] but as yet, few studies have explored the risk factors associated with a positive SARS-CoV-2-positive diagnostic swab test. It is currently not known whether factors indicating increased risk of SARS-CoV-2 infection in adults [18-21], such as exposure history, obesity, lymphocytopenia or ground glass opacity at lung X-ray [19], apply to children. Previous studies in children [22-24] have generally had a small sample size and did not assess predictors of COVID-19 among symptomatic children. 

This study aimed to describe the characteristics of paediatric patients tested for SARS-CoV-2 during the early phase of the pandemic in 20 centres across Italy, and explore factors associated with a positive SARS-CoV-2-positive swab test. 

## Methods

### Study design and participants

This cross-sectional study is reported according to Strengthening the Reporting of Observational studies in Epidemiology (STROBE) guidelines [25]. Data were collected through a collaborative research network coordinated by the World Health Organization (WHO) Collaborating Centre for Maternal and Child Health at the Institute for Maternal and Child Health IRCCS Burlo Garofolo, Trieste, Italy. Children aged 0–18 years tested for SARS-CoV-2 in the period between 23 February and 24 May 2020 in any of the 20 paediatric centres participating in the network were included in the study.

National recommendations on SARS-CoV-2 testing did not change during the study period, and indicated testing for: (i) contacts of COVID-19-positive cases, (ii) cases of severe acute respiratory distress syndrome and (iii) cases with fever, cough or difficulty breathing and absence of another aetiology that could fully explain the clinical presentation [26]. In addition, based on the local epidemiology and on emerging evidence on COVID-19 [27-29], some facilities implemented during the study period local policies of testing all hospitalised children and/or children with gastrointestinal or cutaneous symptoms such as vasculitis.

SARS-CoV-2 infection was diagnosed, in line with national recommendations, using nasal or nasopharyngeal swab specimens collected by trained personnel and tested for SARS-CoV-2 nucleic acid in regional referral laboratories using WHO-recommended real-time RT-PCR assays.

### Data collection and management

Data were collected with a standardised, field-tested online anonymous form, previously used for another study [12] and further optimised and adapted for the purpose of this study. The form collected variables to classify children in the following predefined categories: (i) children tested because of symptoms suggestive of COVID-19, (ii) asymptomatic children tested because of contact with a SARS-CoV-2-positive case and (iii) hospitalised children tested as part of a hospital screening programme. It included information on sociodemographic and clinical characteristics, diagnostic examinations, type of treatments, and outcomes. Both closed and open questions were used. Data were obtained from official medical records and entered in the form by clinical staff in charge of case management in each facility. Information for health workers on how to complete the form was embedded in the form itself. Data collection forms where checked in real time for internal consistency or missing data by trained personnel. Additional cross-checking and data cleaning were conducted before data analysis by two expert biostatisticians (authors IM and BA).

### Study variables

We included in this study sociodemographic and clinical characteristics, diagnostic examinations, type of treatments, and outcomes variables. For children tested because of symptoms suggestive of COVID-19, disease severity was classified using predefined objective criteria adapted from a previously published classification [12] (Supplementary Table S1). Tachypnoea and tachycardia where defined as detailed in Supplementary Table S2. The outcome variable for multivariate analysis was testing positive for SARS-CoV-2.

### Statistical analysis

Categorical variables were reported as absolute numbers and percentages. Continuous variables were expressed as means and standard deviations (SD) or as median and interquartile ranges (IQR), if not normally distributed. We tested for associations between individual covariates and the outcome of a positive SARS-CoV-2 swab using chi-square test or Fisher’s exact test, as appropriate. Variables with a significant univariate relationship with the outcome, available in the whole sample, unless collinear, were included in a generalised estimating equations (GEE) logistic regression model using a compound symmetry covariance structure within centres. The GEE model accounts for correlation between patients who refer to the same centre. We performed separate analyses in three subgroups: (i) children tested because of symptoms suggestive of COVID-19, (ii) asymptomatic children tested because of contact with a case with a SARS-CoV-2-positive test and (iii) hospitalised children tested a s part of a hospital screening programme. The analyses of children tested because of symptoms suggestive of COVID-19 were predefined as our primary analyses, while the analyses in the other two subgroups were considered secondary analyses. An exploratory subgroup analysis was performed on disease severity by age group and sex in patients with symptoms suggestive of COVID-19. We also performed secondary analyses to describe variation across centres in the rate of children with positive SARS-CoV-2 tests. The significance level was set at 0.05 (two-tailed test). Data were analysed with STATA 14 and SAS 9.4.

### Ethical statement

The study was approved by the Institutional Review Board of the Institute for Maternal and Child Health IRCCS Burlo Garofolo, Trieste, Italy (reference number 01/2020 25.03.2020). Data were collected in an anonymous way, analysed and reported only in aggregate form. Given the purely descriptive and retrospective nature of the study, informed consent was waived.

## Results

**Figure 1:**
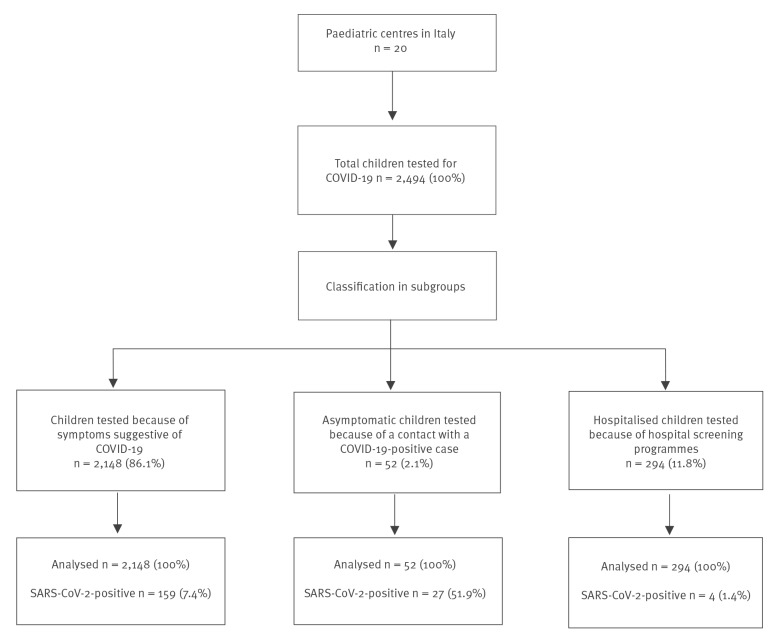
Study flow diagram, paediatric SARS-CoV-2, Italy, 23 February–24 May 2020 (n = 2,494)

During the study period, 2,494 children were tested for SARS-CoV-2 across 20 centres (Figure 1). Geographical case distribution is depicted in Supplementary Figure S1. In the total sample, 2,148 (86.1%) children were tested because of symptoms suggestive of COVID-19, 52 (2.1%) asymptomatic children were tested because of contact with a SARS-CoV-2-positive case and 294 (11.8%) children were tested within hospital screening programmes. Among all the tested children, 190 (7.6%) resulted positive. The percentage of positive cases was significantly higher in those tested because of a SARS-CoV-2-positive contact (COVID-19-positive rate: 51.9%) than in those tested because of symptoms (SARS-CoV-2-positive rate: 7.4%; p < 0.0001) or in hospital screening programmes (SARS-CoV-2-positive rate: 2.1%; p < 0.0001) (Figure 1).

**Figure 2:**
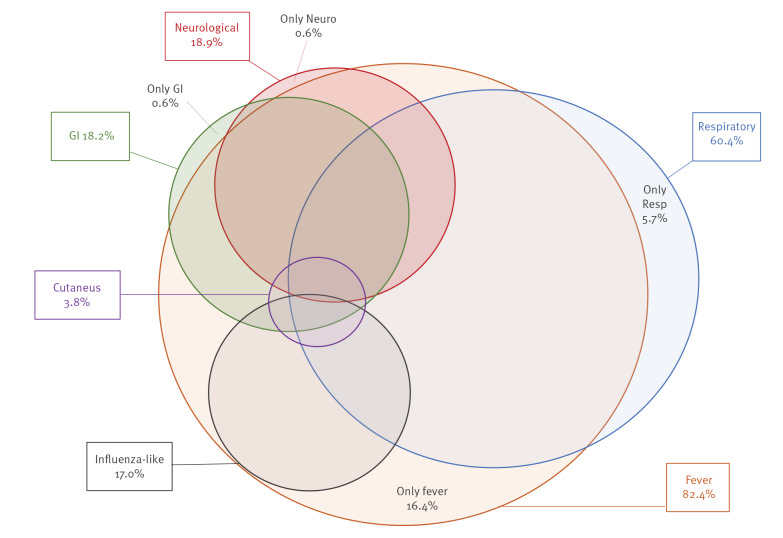
Clinical presentation of SARS-CoV-2-positive children, Italy, 23 February–24 May 2020 (n =159)

GI: gastrointestinal; SARS-CoV-2: severe acute respiratory syndrome coronavirus 2.

The clinical presentations of the 159 SARS-CoV-2-positive cases tested because of symptoms included, besides fever and/or respiratory signs or symptoms, gastrointestinal, neurological and dermatological manifestations and other unspecific influenza-like features (Figure 2). Specifically, 131 children (82.4%) had fever, which presented as the only symptom in 26 (16.4%), and 96 (60.4%) had respiratory signs/symptoms, which presented alone in nine (5.7%). Neurological symptoms such as convulsion, irritability, headache, anosmia/ageusia were observed in 30 (18.9%), in one child as the only symptom. Unspecific general influenza-like symptoms -such as muscular-articular pains, nausea and poor appetite- were reported in 27 (17.0%), always in combination with other clinical signs. Six children were tested because of cutaneous signs such as vasculitis and pseudo-chilblains on fingertips and toes, always in association with other symptoms of any type (fever, respiratory, neurological, unspecific influenza-like symptoms, or gastrointestinal symptoms ).

When comparing children based on the results of swab testing (Table 1), SARS-CoV-2-positive children were more often in the stratum of 10–18 years compared with the SARS-CoV-2-negative (54.1% vs 26.0%; p < 0.001). No difference by sex was observed. A history of SARS-CoV-2-positive contact was strongly associated with an increased risk of a positive swab (79.2% vs 6.1%; p < 0.001). Similarly, having a relative with respiratory symptoms was strongly associated with SARS-CoV-2-positive test results (72.3% vs 11.5%; p < 0.001). Both positive and negative children had a non-negligible rate of comorbidities (17.6% vs 16.4%; p = 0.7), with cardiac diseases being slightly more frequent in the group testing positive for SARS-CoV-2 (5.7% vs 2.1%; p = 0.005).

**Table 1:** Socio-demographic characteristics of children tested for SARS-CoV-2 because of symptoms suggestive of COVID-19, Italy, 23 February–24 May 2020 (n =2,148)

Characteristics	Positive swab n = 159		Negative swab n = 1,989		p value
	n	%	n	%	
Age group					
< 6 months	19	12.0	159	8.0	0.082
6– <24 months	17	10.7	472	23.7	**< 0.001**
2–9 years	37	23.3	836	42.0	**< 0.001**
10–18 years	86	54.1	517	26.0	**< 0.001**
Missing	0	0	5	0.3	1.000
Sex					
Male	77	48.4	1,108	55.7	0.076
Female	82	51.6	880	44.2	0.074
Missing	0	0	1	0.1	1.000
Contact with COVID-19 case	126	79.2	122	6.1	**< 0.001**
Relatives with respiratory symptoms	115	72.3	229	11.5	**< 0.001**
Any comorbidity	28	17.6	327	16.4	0.702
Type of comorbidity					
Malformation, disabilities, neuromuscular diseases	5	3.1	81	4.1	0.566
Cardiac diseases	9	5.7	42	2.1	**0.005**
Asthma	6	3.8	60	3.0	0.593
Other respiratory diseases/conditions	0	0	17	0.9	0.631
Primary immunodeficiencies	1	0.6	12	0.6	1.000
Secondary immunodeficiencies	1	0.6	41	2.1	0.365
Obesity	1	0.6	12	0.6	1.000
Diabetes	1	0.6	2	0.1	0.206
Psychiatric disorders	1	0.6	21	1.1	1.000
Other	9	5.7	97	4.9	0.659

COVID-19: coronavirus disease; SARS-CoV-2: severe acute respiratory syndrome coronavirus 2.

When compared for disease severity at presentation (Table 2), there were no significant differences between children who were SARS-CoV-2-positive and those who were negative. Most cases had a mild presentation (78% and 70.8%, respectively; p = 0.053). Severe (3.1% vs 6.7%; p = 0.091) or critical presentation (1.3% vs 1.0%; p = 0.665) had the same frequency in SARS-CoV-2-positive and those who were negative.

**Table 2:** Clinical presentation and outcomes of children tested for SARS-CoV-2 because of symptoms suggestive of COVID-19, Italy, 23 February–24 May 2020 (n = 2,148)

Clinical presentation and outcomes	Positive swab n = 159		Negative swab n = 1,989		p value
	n	%	n	%	
Disease severity at presentation					
Asymptomatic	8	5.0	125	6.3	0.528
Mild	124	78.0	1,408	70.8	0.053
Moderate	20	12.6	304	15.3	0.359
Severe	5	3.1	133	6.7	0.091
Critical	2	1.3	19	1.0	0.665
Symptoms and signs at presentation					
Fever	131	82.4	1,355	68.1	< **0.001**
Respiratory symptoms, any	96	60.4	1,325	66.6	0.110
Respiratory distress	12	7.5	255	12.8	0.052
Rhinorrhoea	32	20.1	372	18.7	0.659
Dry cough	51	32.1	452	22.7	**0.007**
Productive cough	7	4.4	185	9.3	**0.037**
Sore throat	36	22.6	881	44.3	**< 0.001**
Pharyngitis	2	1.3	106	5.3	**0.024**
Conjunctivitis	8	5.0	60	3.0	0.163
Apnoea	0	0	4	0.2	1.000
Thoracic pain	6	3.8	44	2.2	0.209
Gastrointestinal symptoms, any	29	18.2	574	28.9	0.004
Vomiting	16	10.1	365	18.3	**0.009**
Diarrhoea	18	11.3	293	14.7	0.240
Neurological symptoms, any	30	18.9	175	8.8	**< 0.001**
Asthenia	10	6.3	38	1.9	**< 0.001**
Headache	13	8.2	79	4.0	**0.012**
Anosmia/ageusia	13	8.2	10	0.5	**< 0.001**
Convulsion	2	1.3	49	2.5	0.583
Hyperactivity	1	0.6	12	0.6	1.000
Cutaneous presentations, any	6	3.8	159	8.0	0.054
Skin manifestations	6	3.8	158	7.9	0.057
Vasculitis	0	0	11	0.6	1.000
Unspecific influenza-like presentations, any	27	17.0	303	15.2	0.557
Muscle or joint pains	18	11.3	71	3.6	**< 0.001**
Nausea	0	0	13	0.7	0.616
Inappetence	15	9.4	237	11.9	0.349
Lymphadenitis	8	5.0	158	7.9	0.186
Other symptoms, any	23	14.5	528	26.6	0.001
Abdominal pains	11	6.9	269	13.5	**0.017**
Oral manifestations (gingivostomatitis, aphthae)	2	1.3	54	2.7	0.433
Dental problems	1	0.6	6	0.3	0.417
Urogenital disorders	0	0	10	0.5	1.000
Ear problems	0	0	32	1.6	0.166
Others	3	1.9	41	2.1	1.000
Vital parameters at presentation					
Tachycardia	12/61	19.7	294/1,489	19.7	0.989
Tachypnoea	4/34	11.8	187/827	22.6	0.204
Oxygen saturation level at presentation					
91–92%	2/66	3.0	15/1,575	1.0	0.147
≤ 90%	1/66	1.5	21/1,575	1.3	0.597
Clinical examination at presentation					
Lung auscultation					
Negative	69/86	80.2	1,486/1,826	81.3	0.810
Crackles	4/86	4.7	186/1,826	10.2	0.099
Wheezing	3/86	3.5	120/1,826	5.6	0.366
Absent breath sounds	4/86	4.7	111/1,826	6.1	0.816
Laboratory test^a^					
White blood cell count < 5.5 (× 10^9^/L)	17/50	34.0	109/809	13.5	**< 0.001**
Lymphocyte count < 1.2 (× 10^9^/L)	8/41	19.5	75/559	13.4	0.275
Neutrophil < 1.50 (× 10^9^/L)	6/47	12.8	50/743	6.7	0.118
C-reactive protein > 1 gr/dL	29/50	58.0	589/760	77.5	**0.002**
Erythrocyte sedimentation rate > 20 mm/h	2/4	50.0	34/64	53.1	1.000
Aspartate aminotransferase > 50 (U/L)	7/35	20.0	56/434	12.9	0.234
Alanine aminotransferase > 45 (U/L)	4/46	8.7	74/692	10.7	0.808
D dimer > 0.5 (μg/mL)	2/4	50.0	24/46	52.2	1.000
Chest X-ray	27	17.0	313	15.7	0.679
Negative	8/27	29.6	106/313	33.9	0.655
Ground glass opacities	7/27	25.9	71/313	22.7	0.701
Focal consolidation	3/27	11.1	77/313	24.6	0.155
Other description	9/27	33.3	59/313	18.8	0.071
Lung ultrasound	5	3.1	58	2.9	0.806
Negative	1/5	20.0	18/58	31.0	1.000
B-lines in various pattern	2/5	40.0	30/58	51.7	0.672
Focal consolidation	0	0	5/58	8.6	1.000
Other description	1/5	20.0	1/58	1.7	0.154
Lung CT scan	5	3.1	13	0.7	0.008
Negative	0/5	0	2/13	15.4	1.000
Ground glass opacities	3/5	60.0	6/13	46.2	1.000
Focal consolidation	1/5	20.0	3/13	23.1	1.000
Other description	0/5	0	2/13	15.4	1.000
Hospitalised	45	28.3	602	30.3	0.603
Respiratory support^a^	4	2.5	73	3.7	0.656
Oxygen	3/45	6.7	54/602	9.0	0.788
High flow oxygen	2/45	4.4	19/602	3.2	0.651
Non-invasive ventilation	1/45	2.2	4/602	0.7	0.303
Mechanical ventilation	0/45	0	11/602	1.8	1.000
Cases in ICU	2	1.3	11	0.6	0.250
Outcome					
Cured	159	100	1,981	99.6	0.500
Referred	0	0	7	0.4	0.460
Died	0	0	1	0.1	1.000

COVID-19: coronavirus disease; ICU: intensive care unit; SARS-CoV-2: severe acute respiratory syndrome coronavirus 2.

^a^ Available in a subsample of cases.

Fever was highly prevalent in both groups, significantly more in SARS-CoV-2-positive children (82.4% vs 68.1%, p < 0.001). Respiratory symptoms were highly prevalent in both groups (60.4% vs 66.6%; p = 0.110) with some differences: dry cough was more frequent in the group of SARS-CoV-2-positive individuals (32.1% vs 22.7%; p = 0.007), whereas sore throat and pharyngitis, were less frequent (22.6% vs 44.3%; p < 0.001 and 1.3% vs 5.3%; p = 0.024 respectively). Respiratory distress was less frequent in the group testing positive for SARS-CoV-2 than in the group testing negative, although the difference was not statistically significant (7.5% vs 12.8%; p = 0.052). Gastrointestinal symptoms (18.2% vs 28.9%; p = 0.004) and other symptoms (14.5% vs 26.6%; p = 0.001) were significantly less frequent in the SARS-CoV-2-positive group, while the opposite was true of neurological symptoms (18.9% vs 8.8%; p < 0.001) and muscle or joint pains (11.3% vs 3.6%; p < 0.001).

Vital parameters as well as oxygen saturation levels and lung auscultation were not significantly different between the two groups. Lymphocytopenia was significantly more frequent in the SARS-CoV-2-positive group (34.0% vs 13.5%; p < 0.001), while elevated C-reactive protein was more frequent in the SARS-CoV-2-negative group (58.0% vs 77.5%; p = 0.002). Findings at chest-X-ray, lung ultrasound and lung computed tomography scan were not significantly different between the two groups, with equal prevalence of ground glass opacities in SARS-CoV-2-positive and those who were negative (respectively 25.9% vs 22.7%; p = 0.7 and 40.0% vs 51.7%; p = 0.67).

The frequencies of hospitalised cases (28.3% vs 30.3%; p = 0.60) and those admitted to an intensive care unit (ICU) (1.3% vs 0.6%; p = 0.25) were not significantly different between the SARS-CoV-2-positive and -negative children. Need and type of respiratory support were also not significantly different. Final outcomes did not differ between groups, although one death occurred in the SARS-CoV-2-negative group.

### Multivariate analysis

In multivariate analysis, factors significantly associated with testing positive for SARS-CoV-2 were: contact with COVID-19-positive patient (odds ratio (OR): 39.83; 95% confidence interval (CI): 17.52–90.55; p < 0.0001), pre-existing cardiac disease (OR: 3.10; 95% CI: 1.19–5.02; p < 0.0001), fever (OR: 3.05%; 95% CI: 1.67–5.58; p = 0.0003) and anosmia/ageusia (OR: 4.08; 95% CI: 1.69–9.84; p = 0.002) (Table 3). Age between 2 and 9 years was negatively associated with testing positive for COVID-19, when taking the group of 10–18 years as reference (OR: 0.33; 95% CI: 0.22–0.50; p < 0.0001).

**Table 3:** Multivariate analysis of characteristics and risk indicators for SARS-CoV-2 in children, Italy, 23 February–24 May 2020 (n = 2,148)

Characteristics	Adjusted OR (95% CI)	p value
Age group		
< 6 months	1.12 (0.60–2.11)	0.725
6– <24 months	0.43 (0.15–1.20)	0.107
2–9 years	0.33 (0.22–0.50)	**< 0.0001**
10–18 years	Reference	
Risk indicator		
Contact with SARS-CoV-2-positive case	39.83 (17.52–90.55)	**< 0.0001**
Cardiac disease	3.10 (1.19–5.02)	**< 0.0001**
Fever	3.05 (1.67–5.58)	**0.0003**
Dry cough	1.31 (0.87–2.01)	0.199
Productive cough	0.53 (0.18–1.53)	0.242
Sore throat	0.54 (0.29–1.03)	0.063
Pharyngitis	0.41 (0.09–1.86)	0.246
Vomiting	1.01 (0.68–1.50)	0.963
Asthenia	0.94 (0.31–2.84)	0.911
Headache	0.98 (0.52–1.87)	0.956
Anosmia/ageusia	4.08 (1.69–9.84)	**0.002**
Muscle or joint pain	1.76 (0.86–3.63)	0.124
Abdominal pains	1.05 (0.57–1.94)	0.882

CI: confidence interval; OR: odds ratio; SARS-CoV-2: severe acute respiratory syndrome coronavirus 2.

### Secondary analyses

Additional details on the 190 children positive for SARS-CoV-2 are reported in Supplementary Table S3. Overall, 139 (73.1%) children were cared for at home. The remaining were hospitalised in two types of wards: paediatric wards (n = 25; 55.6%) and general COVID-19 wards (n = 24; 53.3%). Cases treated with home care were either asymptomatic or had a mild or moderate presentation. The children received different types of treatments; antibiotics, steroids and hydroxychloroquine were more frequently prescribed among hospitalised children (respectively 35.6% vs 10.5%; p < 0.001, 8.9% vs 0.0%; p = 0.006 and 8.9% vs 0.0%; p = 0.006), while antipyretics/analgesic were much more frequently used in home care management (2.2% vs 51.8%; p < 0.001).

Sociodemographic data, clinical characteristics and outcomes of children tested because of a COVID-19-positive contact and of those tested through hospital screening are reported in Supplementary Table S4. No significant difference was observed for any variable between the SARS-CoV-2-positive individuals and the negatives in these two groups. None of the children positive for SARS-CoV-2 in these groups had respiratory distress, none required respiratory support, none were admitted to ICU and all recovered.

No difference in disease severity was observed by age and sex, in SARS-CoV-2-positive children (n = 159) (Table 4).

**Table 4:** Disease severity by sex and age in SARS-CoV-2-positive children with symptoms suggestive of COVID-19, Italy, 23 February–24 May 2020 (n = 159)

Disease severity	Sex									
	Male n = 77					Female n = 82				
	n			%		n			%	
Asymptomatic	4			**5.2**		4			**4.9**	
Mild	56			72.7		68			82.9	
Moderate	13			16.9		7			8.5	
Severe	3			3.9		2			2.4	
Critical	1			1.3		1			1.2	
**Disease severity**	**Age**									
	**< 6 months** **n = 19**		**6– <24 months** **n = 17**				**2–9 years** **n = 37**		**10–18 years** **n = 86**	
	**n**	**%**	**n**		**%**		**n**	**%**	**n**	**%**
Asymptomatic	3	15.8	1		5.9		2	5.4	2	2.3
Mild	11	57.9	14		82.4		32	86.5	67	77.9
Moderate	3	15.8	1		5.9		1	2.7	15	17.4
Severe	1	5.3	1		5.9		1	2.7	2	2.3
Critical	1	5.3	0		0		1	2.7	0	0

COVID-19: coronavirus disease; ICU: intensive care unit; SARS-CoV-2: severe acute respiratory syndrome coronavirus 2.

There was no significant difference (p < 0.05) by age and sex.

The rate of children testing positive for SARS-CoV-2 at each centre was variable with an average rate of 9.17% (95% CI: 0–18.71; Supplementary Figure S2). Specifically, in 17 of 20 centres, the prevalence of COVID-19-positive swabs was below 15%, in two centres, it was in between 15% and 20% and in one centre, it was over 20%.

## Discussion

This study adds to previous knowledge a description of characteristics and risk factors for SARS-CoV-2 among children from the early stages of the COVID-19 pandemic in Europe. Notably, the clinical presentation of children with SARS-CoV-2 includes different possible scenarios. Besides the typical clinical picture with fever and respiratory signs or symptoms, this study suggested that COVID-19 in children may have neurological, gastrointestinal, or cutaneous presentations, either in combination with other presentations or alone. These results are in line with reports from rheumatologists and dermatologists [27,28], gastroenterologists [29], neurologists and psychiatrists [30,31]. Although the case definition of ‘suspected case of SARS-CoV-2 infection’ was updated by the WHO in December 2020 [32], it does not yet include all possible clinical presentations of COVID-19, as highlighted by this study and other evidence in the literature [27-31]. Findings from COVID-19 screening among categories of people at risk, such as health workers, indicate that the current guidelines [26] for testing may risk missing many cases [33]. Furthermore, it is important to acknowledge that, because the recommendations on SARS-CoV-2 case identification at the time of this study indicated testing only for cases with either fever or respiratory signs [26,34], the real prevalence of other presentations (e.g. gastrointestinal, neurological and cutaneous) may have gone underestimated in this study, as well as in other studies. Guidelines for SARS-CoV-2 testing should be updated based on the evidence on clinical presentation of the disease in children and adults.

Our findings suggest that, in contrast with what has been observed in adults [18,19], there are very few features in children which help differentiate those affected by SARS-CoV-2 from those with other conditions. Specifically, some of the features identified so far in the few existing studies as predicting factors for COVID-19 in adults, such as obesity, leukopenia, lymphocytopenia, ground glass opacity at X-ray and having both lungs affected [19], were not confirmed in children. This seems plausible, considering the generally mild presentation of SARS-CoV-2 in the paediatric age range and the large number of other viruses which can affect children and result in clinical pictures very similar to COVID-19.

Our findings indicate that a diagnosis of SARS-CoV-2 may be much more probable in those who had contact with a person testing positive for SARS-CoV-2 (OR: 39.83; 95% CI: 17.52–90.55) or in children with fever (OR: 3.05; 95% CI: 1.67–5.58) or anosmia/ageusia (OR: 4.08; 95 %CI 1.69–9.84; p = 0.002). These results are in line with studies in adults [19,30] and underscore the importance of testing all cases with exposure history and increased body temperature, as well as those with peculiar neurological signs.

Our findings related to young age as a protective factor (with children in the age range 2–9 years being at lower risk of COVID-19 compared with the reference group of 10–18-year-olds: OR: 0.33; 95% CI: 0.22–0.50) and to presence of cardiac disease as a risk factor (OR: 3.10; 95% CI: 1.19–5.02) are novel and warrant further confirmation and identification of causal mechanisms. Interestingly, about one in six children accessing the health system with a presentation suggestive of COVID-19 had a comorbidity (355/2,148; 16.5%). Nevertheless, the only comorbidity associated with positive testing for SARS-CoV-2 was pre-existing cardiac disease. Interestingly, pre-existing chronic kidney disease was a significant predictive factor for COVID-19 diagnosis in one large study at the primary care level in England, not specific to children [18]. These results should be confirmed in larger studies in children. More studies should explore if other factors apparently important in adults [18] – such as ethnicity, living situation, deprivation, children with smoking parents or obesity – increase the risk of COVID-19 in children.

This study suggests that COVID-19 has been a mild disease in children in Italy: among the 190 children diagnosed with SARS-CoV-2 in our study, 12 (6.3%) had respiratory distress, only four (2.1%) required respiratory support, only two (1.1%) were admitted to ICU and all of them recovered. These results are in line with surveillance data in Italy [35], and with previous reports on COVID-19 in children from different countries [4-6,16,36,37]. According to existing surveillance data from the United States (US) Centers for Disease Control and Prevention, the number of deaths among children under 15 years of age with COVID-19 in the United States was much lower than what was reported for children with seasonal influenzas in 2019/20 (17 reported deaths for COVID-19 compared with 182 influenza-associated paediatric deaths) [38,39]. In contrast, data from adults indicate that COVID-19 may be more severe than influenzas in this population [40].

The sample of children hospitalised in this study was small (51 cases) but not negligible when compared with national data at the time of the study: by 20 May 2020, the Italian surveillance systems had reported 227,204 confirmed SARS-CoV-2 cases in Italy, but only 123 hospitalised cases among children (i.e. age below 18 years) [35]. Our sample of 51 children hospitalised with COVID-19 thus accounts for 41.4% of the total paediatric cases reported by national surveillance [35]. Clearly, larger prevalence studies as well as prospective longitudinal studies are needed to better understand the risk associated with COVID-19 in selected subpopulations of children at risk. Despite current preliminary evidence suggesting that even in children with underlying conditions – such as inflammatory bowel diseases [41], cancer [42], dialysis [43] and renal diseases with steroid treatment [44] – the risk of severe COVID-19 disease may be limited, much more solid data are needed.

This study highlights several interesting epidemiological findings, reporting the number of children tested in several centres in the early phase of the pandemic and the rate of positivity. High heterogeneity across centres in the rate of positive SARS-CoV-2 testing is not surprising and may have multiple explanations. Firstly, the epidemiology of the disease differs across Italy, where regions in the north overall had a higher burden of cases compared with those in the south [45]. Secondly, case identification may have been affected by local protocols, testing capacities and different implementation of testing recommendations, both at study start and over time. The number of total swabs per population has been reported as highly variable across regions in Italy and not always directly proportional to the incidence of COVID-19 disease, with considerable variations over time [46]. The implementation of case finding and contact tracing has been described as highly heterogenous in other countries [47] and would warrant further investigation to better interpret epidemiological curves. Epidemiological data on COVID-19 may not reflect the real incidence of the disease in each setting, partly because of limitations in the currently available technology for COVID-19 diagnosis (i.e. high rates of false negatives with nasal or nasopharyngeal swabs [48]); it should, in general, be interpreted with extreme caution. Further studies should document knowledge, attitudes and practices of case finding and contact tracing. More accurate, acceptable and sustainable tools are also needed for COVID-19 diagnosis.

Limitations of this study include the retrospective nature of data, possible selection bias towards more symptomatic cases owing to the nature of the network, and the limitation in the technology currently available for COVID-19 diagnosis. Although the use of swabs is currently recommended as the gold standard for COVID-19 diagnosis, it has as major limitation of a high percentage of false negative cases [48]. Future studies, when better diagnostic tools will be available, should aim to confirm the observations of the present study. Strengths of this study include its pragmatic and descriptive nature, and the involvement of many paediatric centres in the national territory. More clinical and epidemiological studies are needed to further document the real incidence, presentation, risk factors and outcomes of children with COVID-19 infection in different paediatric subpopulations, to better characterise children at higher risk of the most severe forms of the disease.

## Supplementary Data

275000 Supplement
