# Supplementary material for: Characteristics and risk factors for SARS-CoV-2 in children tested in the early phase of the pandemic: a cross-sectional study, Italy, 23 February to 24 May 2020
Source: Euro Surveill. 2021 Apr 8;26(14):2001248. doi: 10.2807/1560-7917.ES.2021.26.14.2001248 (PMC8034058; doi:10.2807/1560-7917.ES.2021.26.14.2001248)

Supplementary file

Characteristics and risk factors for SARS-CoV-2 in children tested in the early phase of the pandemic: a cross-sectional study, Italy, 23 February to 24 May 2020

This supplementary material is hosted by *Eurosurveillance* as supporting information alongside the article **Characteristics and risk factors for SARS-CoV-2 in children tested in the early phase of the pandemic: a cross-sectional study, Italy, 23 February to 24 May 2020**, on behalf of the authors, who remain responsible for the accuracy and appropriateness of the content. The same standards for ethics, copyright, attributions and permissions as for the article apply. Supplements are not edited by *Eurosurveillance* and the journal is not responsible for the maintenance of any links or email addresses provided therein.

Table of content

Supplementary Table 1. Criteria for disease severity 2

Supplementary Table 2. Definition of tachypnea and tachycardia 3

Supplementary Table 3. Treatments and outcomes of children SARS-CoV-2 positive 4

Supplementary Table 4. Characteristics of children tested for SARS-COv-2 because of contact with a positive case or within hospitals screening 5

Supplementary Figure 1. Geographical distribution of total cases (N=2494) and positive cases (N=190) 8

Supplementary Figure 2. Percentage of SARS-CoV-2 positive cases across centers 9

Supplementary Table 1. Criteria for disease severity

Asymptomatic: all the following must be present

1. No signs or symptoms
2. AND Negative Chest X-Ray
3. AND absence of criteria for other cases

Mild: any of the following (AND absence of criteria for more severe cases)

1. Symptoms of upper respiratory tract infection
2. AND absence of pneumonia at chest X-ray

Moderate: all the following (AND absence of criteria for more severe cases)

1. Cough or other symptom/sign AND pneumonia at chest X-ray
2. CT with lung lesions

Severe: any of the following (AND absence of criteria as for critical case)

1. Oxygen saturation <92% OR
2. Difficult breathing or other signs of severe respiratory distress (apnea, gasping, head nodding) or any other danger sign such as convulsion, restlessness, lethargy, severe dehydration, reduced reactivity, hypotonia
3. OR need for any respiratory support

Critical: Any of the following

1. Patient in ICU
2. OR mechanical ventilation
3. OR multiorgan failure
4. OR shock, encephalopathy, myocardial injury or heart failure, coagulation disfunction, acute kidney injury, coma.

Abbreviations: CT= Computed tomography; ICU = Intensive Care Unit;

Reference: Parri N, Magistà AM, Marchetti F, et al. Characteristic of COVID-19 infection in pediatric patients: early findings from two Italian Pediatric Research Networks [published online ahead of print, 2020 Jun 3]. Eur J Pediatr. 2020;1-9. doi:10.1007/s00431-020-03683-8 (adapted)

Supplementary Table 2. Definition of tachypnea and tachycardia

**Tachypnea**	
Age	Respiratory rate (breaths/min)
2 months	60
2-12 months	50
1-5 years	40
>5 years	30
**Tachycardia**	
Age	Heart rate (beats per min)
Term	>170
3-12 months	>165
1-5 years	>150
6-11 years	>135
>11 years	>120

Supplementary Table 3. Treatments and outcomes of children SARS-CoV-2 positive

	Groups of children					
	Symptoms suggestive for COVID-19 (N=159)		Contact with COVID-19 N=27		Screening for hospitalisation N=4	
	Hospitalised N=45 n (%)	Home care N=114 n (%)	Hospitalised N=3 n (%)	Home care N=24 n (%)	Hospitalised N=3 n (%)	Home care N=1 ** n (%)
**Type of hospitalization***						
Pediatric ward	25 (55.6)	-	2 (66.7)	-	1	-
COVID-19 ward	24 (53.3)	-	2 (66.7)	-	0	-
In observation	6 (13.3)	-	0	-	1 (33.3)	-
Infectious diseases ward	5 (11.1)	-	0	-	0	-
Intensive care unit	2 (4.4)	-	0	-	0	-
**Type of respiratory support any**	4 (2.5)	-	0	-	0	-
Oxygen	3 (6.7)	-	0	-	0	-
High flow oxygen	2 (4.4)	-	0	-	0	-
Non-invasive ventilation	1 (2.2)	-	0	-	0	-
Mechanical ventilation	0	-	0	-	0	-
**Clinical course**						
Asymptomatic	6 (13.3)[1]	2 (1.8)	3 (100)	24 (100)	2 (66.7)	1 (100)
Mild	21 (46.7)[1]	103 (90.4)	0	0	0	0
Moderate	11 (24.4)[1]	9 (7.9)	0	0	0	0
Severe	5 (11.1)[1]	0	0	0	1 (33.3)	0
Critical	2 (4.4)	0	0	0	0	0
**Treatment**						
Antibiotic	16 (35.6)[1]	12 (10.5)	0	0	0	0
Hydroxychloroquine	4 (8.9)[1]	0	0	0	0	0
Steroids	4 (8.9)[1]	0	0	0	0	0
Anticoagulant	2 (4.4)	0	0	0	0	0
Antiviral	2 (4.4)	0	0	0	0	0
Adrenaline	2 (4.4)	0	0	0	0	0
Immunoglobulin	2 (4.4)	0	0	0	0	0
Antipyretics/analgesic	1 (2.2)[1]	59 (51.8)	0	0	0	0
Others drugs	1 (2.2)	12 (10.5)	0	1 (4.2)	0	0
Antiepileptic	0	1 (0.9)	0	0	0	0
**Final outcome**						
Cured	45 (100)	114 (100)	3 (100)	24 (100)	3 (100)	1 (100)
Referred	0	0	0	0	0	0
Dead	0	0	0	0	0	0

[1] Significant difference ($p<0.05$) between hospitalised and home groups

* Total percentage exceeds 100% because some patients are double counted since moved from a ward to another.

** Child screened because of a day hospital

Abbreviations and symbols: IQR = inter-quartile ranges; - = not applicable.

Supplementary Table 4. Characteristics of children tested for SARS-CoV-2 because of contact with a positive case or within hospitals screening

	Contact with COVID-19 N=52		Screening for hospitalisation N=294	
	**Positive swab N=27 n (%)**	**Negative swab N=25 n (%)**	**Positive swab N=4 n (%)**	**Negative swab N=290 n (%)**
**Age range**	0-17	0-17	0-12	0-18
**Age groups**				
< 6 months	2 (7.4)	3 (12.0)	1 (25.0)	28 (9.7)
6-24 months	2 (7.4)	1 (4.0)	1 (25.0)	36 (12.4)
2-9 years	10 (37.0)	11 (44.0)	1 (25.0)	123 (42.4)
10-18 years	13 (48.1)	10 (40.0)	1 (25.0)	99 (34.1)
Missing	0	0	0	4 (1.4)
**Sex**				
Male	11 (40.7)	11 (44.0)	1 (25.0)	186 (64.1)
Female	16 (59.3)	14 (56.0)	3 (75.0)	100 (34.5)
Missing	0	0	0	4 (1.4)
**Contact with COVID-19 positive**	27 (100)	25 (100)	0	3 (3.0)
**Relatives with respiratory symptoms**	18 (66.7)	19 (76.0)	0	5 (1.7)
**Any co-morbidity**	4 (14.8)	1 (4.0)	0	49 (16.9)
**Type of comorbidities**				
Malformation, disability, neuromuscular diseases	0	0	0	13 (4.5)
Cardiac disease	0	0	0	5 (1.7)
Respiratory disease	0	0	0	1 (0.3)
Asthma	1 (3.7)	0	0	0
Primary immunodeficiency	0	0	0	1 (0.3)
Secondary immunodeficiency	0	0	0	1 (0.3)
Obesity	2 (7.4)	0	0	0
Diabetes	0	0	0	3 (1.0)
Psychiatric disorder	0	0	0	9 (3.1)
Other	1 (3.7)	1 (4.0)	0	19 (6.6)
**Disease severity at presentation**				
Asymptomatic	27 (100)	25 (100)	3 (75.0)	135 (46.6)
Mild	0	0	0	83 (28.6)
Moderate	0	0	0	44 (15.2)
Severe	0	0	1 (25.0)	25 (8.6)
Critical	0	0	0	3 (1.0)
**Symptoms and signs at presentation**				
Fever	0	0	0	22 (7.6)
**Respiratory symptoms, any**	0	0	0	14 (4.8)
Respiratory distress	0	0	0	5 (1.7)
Rhinorrhea	0	0	0	4 (1.4)
Dry cough	0	0	0	2 (0.7)
Productive cough	0	0	0	0
Sore throat	0	0	0	2 (0.7)
Strep throat	0	0	0	0
Conjunctivitis	0	0	0	2 (0.7)
Apnea	0	0	0	0
Thoracic pain	0	0	0	2 (0.7)
**Gastrointestinal symptoms, any**	0	0	0	19 (6.6)
Vomiting	0	0	0	15 (5.2)
Diarrhea	0	0	0	8 (2.8)
**Neurological symptoms, any**	0	0	1 (25.0)	18 (6.2)

Asthenia	0	0	0	2 (0.7)
Headache	0	0	0	7 (2.4)
Anosmia/ageusia	0	0	0	0
Convulsion	0	0	1 (25.0)	7 (2.4)
Hyperactivity	0	0	0	2 (0.7)
**Cutaneous symptoms, any**	0	0	0	2 (0.7)
Skin manifestations	0	0	0	2 (0.7)
Vasculitis	0	0	0	0
**Unspecific flu-like symptoms, any**	0	0	0	18 (6.2)
Muscle or joint pain	0	0	0	8 (2.8)
Nausea	0	0	0	3 (1.0)
Inappetence	0	0	0	10 (3.4)
Lymphadenitis	0	0	0	2 (0.7)
**Other symptoms, any**	0	0	0	61 (21.0)
Abdominal pains	0	0	0	40 (13.8)
Oral manifestations (gingivostomatitis, aphthae)	0	0	0	0
Dental problems	0	0	0	1 (0.3)
Urogenital disorders	0	0	0	10 (3.4)
Ear problems	0	0	0	1 (0.3)
Others	0	0	0	11 (3.8)
**Vital parameters at presentation**				
Tachycardia	0/5	0/5	0/1	15/142 (10.6)
Tachypnea	0/4	0/3	0/1	2/56 (3.6)
**Oxygen saturation level at presentation**				
91-92	0/5	0/7	0/1	0/121
≤90	0/5	0/7	0/1	0/121
**Clinical examination at presentation**				
**Lungs auscultations**				
Negative	7/9 (77.8)	13/13 (100)	1/1 (100)	211/216 (97.7)
Crackles	0/9	0/13	0/1	2/216 (0.9)
Wheezing	0/9	0/13	0/1	0/216
Absent breath sounds	0/9	0/13	0/1	1/216 (0.5)
**Laboratory test**[1]				
White blood cell count <5.5 (×109/L)	0/2	0/1	0/1	2/62 (96.8)
Lymphocyte count <1.2 (×109/L)	0/1	0/1	0	3/36 (8.3)
Neutrophil <1.50 (×109/L)	0/2	0/1	0/1	3/57 (94.7)
C-reactive protein > 1 gr/dl	0/2	0/1	0/1	22/52 (42.3)
Erythrocyte sedimentation rate > 20 mm/h	0	0	0	3/5 (60.0)
Aspartate aminotransferase > 50 (U/L)	0/1	0/1	0	3/23 (13.0)
Alanine aminotransferase > 45 (U/L)	2/2 (100)	0/1	0/1	4/49 (8.2)
D dimer >0.5 (μg/mL)	0	0	0	1/1 (100)
**Chest X-ray**	0	1 (4.0)	0	8 (2.8)
Ground glass opacities [1]	0	0	0	3/8 (37.5)
Negative [1]	0	1 (4.0)	0	4/8 (50.0)
Focal consolidation [1]	0	0	0	1/8 (12.5)
Other description [1]	0	0	0	0/8
**Lung ultrasound**	1 (3.7)	0	0	0
Ground glass opacities [1]	0	0	0	0
Negative [1]	1 (3.7)	0	0	0
Focal consolidation [1]	0	0	0	0
Other description [1]	0	0	0	0
**CT scan**	0	0	1 (25.0)	0

Ground glass opacities [1]	0	0	1/1 (100)	0
**Hospitalised[2]**	3 (11.1)	0	3 (75.0)	272 (93.8)
**Respiratory support[1]**	0	0	0	2 (0.7)
Oxygen	0/3	0/0	0/3	0/272
High flow oxygen	0/3	0/0	0/3	0/272
Noninvasive ventilation	0/3	0/0	0/3	1/272 (0.4)
Mechanical ventilation	0/3	0/0	0/3	2/272 (0.7)
**Cases in ICU**	0/3	0/0	0/3	2/272 (0.7)
**Outcome**				
Cured	27 (100)	25 (100)	4 (100)	289 (99.7)
Referred	0	0	0	1(0.3)
Dead	0	0	0	0

No significant difference ($p<0.05$) between positive and negative swab in each group.

[1] Note: available in a subsample of cases

[2] Note: one child with social problem, one child with burns, one newborn

Supplementary Figure 1. Geographical distribution of total cases (N=2494) and positive cases (N=190)

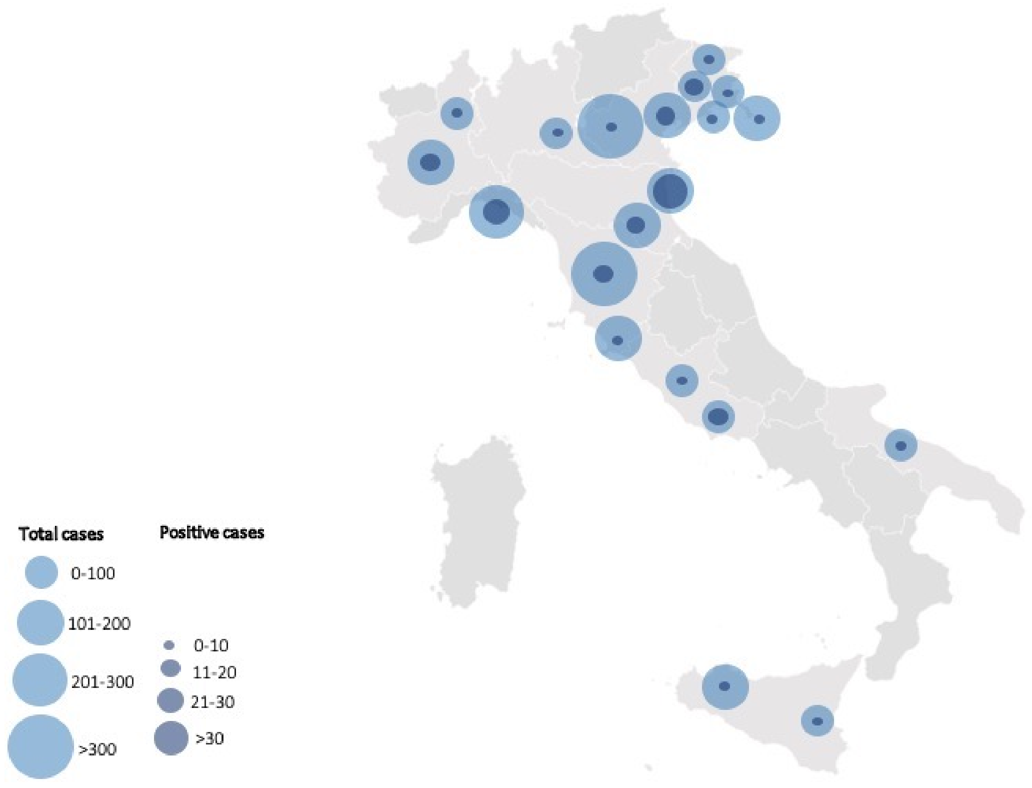

Supplementary Figure 2. Percentage of SARS-CoV-2 positive cases across centers

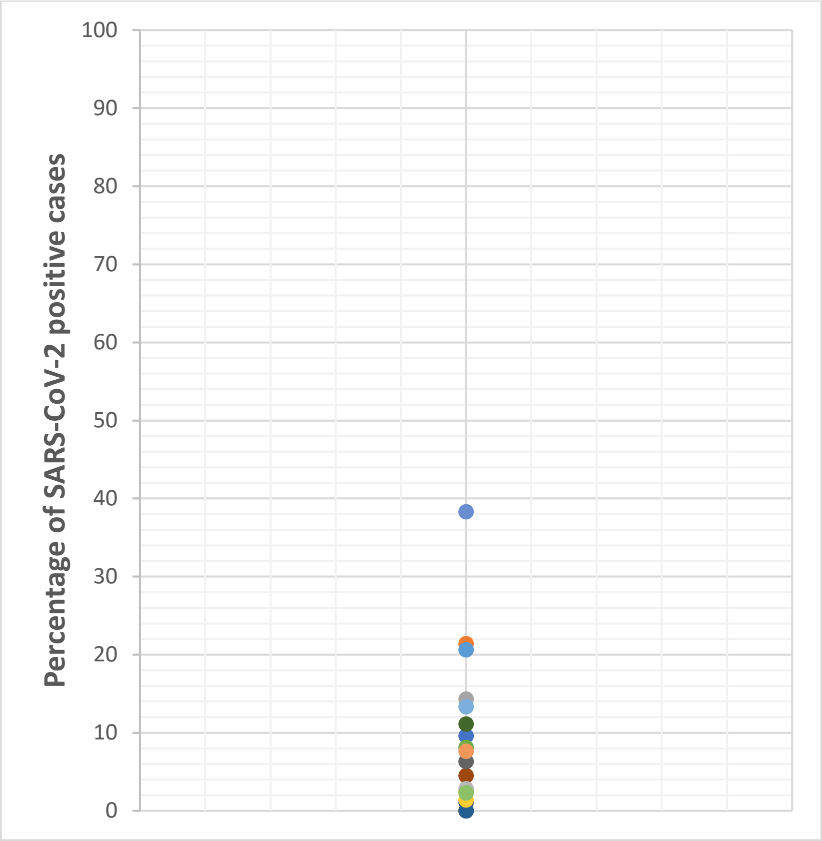

Supplement: Supplement [file 20-01248_LAZZERINI_Supplement.pdf]
